# miR‐1246 is implicated as a possible candidate for endometrium remodelling facilitating implantation in buffalo (*Bubalus bubalis*)

**DOI:** 10.1002/vms3.968

**Published:** 2022-10-25

**Authors:** Pratiksha Dubey, Vipul Batra, Parul Sarwalia, Samiksha Nayak, Rubina Baithalu, Rakesh Kumar, Tirtha Kumar Datta

**Affiliations:** ^1^ Animal Genomiccs Lab, Animal Biotechnology Centre ICAR‐National Dairy Research Institute Karnal India; ^2^ Department of Biological Sciences Indian Institute of Science Education and Research Mohali India; ^3^ ICAR‐Central Institute for Research on Buffaloes Hisar Haryana India

**Keywords:** buffalo, early pregnancy detection, implantation, maternal recognition of pregnancy, miRNA expression

## Abstract

**Background:**

The microRNAs (miRs) secreted by the trophectoderm (TE) cells have recently been implicated in the conceptus‐endometrial cross talk during implantation and placentation. These miRs modulate various cellular processes during conception and throughout the pregnancy by regulating the gene expression in the foetal and maternal tissues.

**Objectives:**

This study was undertaken to elucidate the function of TE secreted miRNAs in the maternal‐foetal cross‐talk during implantation/placentation in buffalo.

**Methods:**

The in vitro produced blastocysts were cultured on a cumulus feeder layer for 21 days. The relative expression profiles of a selected panel of miRs was generated using the spent media collected on Days 0, 7, 12, 16, and 21. A custom‐designed mirVana™ miRNA mimic was used to transfect the endometrial epithelial cells (EECs) in order to determine the role of miRNA exhibiting highest expression on Days 21 and 21.

**Results:**

The expression of miR‐1246 (*p* < 0.001) and let‐7b (*p* < 0.01) was found to be significantly higher on Day 21 of TE culture in comparison to the control (Day 0). This elevated expression indicated the involvement of these miRs in the maternal‐foetal cross‐talk. Interestingly, after the transfection of EECs with miRNA mimic for miR‐1246 (a novel molecule vis‐à‐vis implantation), the expression of beta‐catenin and mucin1 in these cells was found to be significantly (*p* < 0.05) downregulated vis‐à‐vis the control, that is, the IFN‐τ primed EECs (before transfection).

**Conclusions:**

The TE secreted miR‐1246 appeared to lower the expression of the endometrial receptivity genes (mucin1 and beta‐catenin) which apparently assists the endometrium in preparing for placentation.

## Graphical abstract

This study was undertaken to elucidate the function of TE secreted miRNAs in the maternal‐fetal cross‐talk during implantation/placentation in buffalo. The transfection of TE secreted miR‐1246 in endometrial cells appeared to lower the expression of the endometrial receptivity genes (mucin1 and beta‐catenin) which apparently assists the endometrium in preparing for placentation.

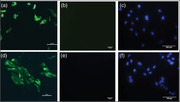

## 1 INTRODUCTION

The recognition and maintenance of pregnancy depend on a series of highly coordinated cellular processes that result in endometrial transformation, conceptus development, implantation and placenta formation (Patterson et al., 2017). The changes in the uterine morphology and physiology are manifested as a result of the bidirectional conceptus‐endometrial cross‐talk. This embryo‐maternal dialogue is a complex process and requires the coordination of both the soluble and insoluble, embryo‐derived factors, for example, the Maternal Recognition of Pregnancy (MRP) agents and endometrial secreted molecules, for example, chemokines, cytokines, adhesion molecules and growth factors that prepare the uterus for implantation (Bazer, 2013; Patterson et al., 2017; Bidarimath and Tayade, 2017). The conceptus secreted MRP agents act on the corpus luteum for continuous production of progesterone which is required for the maintenance of pregnancy. The identity of these agents is species‐specific, for example, interferon tau in ruminants, oestrogen in pigs, and human chorionic gonadotropin (hCG) in humans.

A crucial consequence of the conceptus‐endometrial cross‐talk is implantation, a process which occurs during a restricted period known as the window of implantation. The synchronisation between the development of the embryo (to the blastocyst stage) and differentiation of the uterus (to the receptive state) is deemed essential for successful implantation (Sharma & Kumar, 2012). The endometrium becomes receptive to the conceptus by expressing the receptor molecules for intercellular interaction, for example, integrin, cadherin and osteopontin which help the blastocyst to attach to the endometrium (Haeger et al., 2018). Recently, it has been discovered that the microRNAs (miRNAs or miRs), for example, the ones secreted by trophoblast cells play a crucial role in the embryo‐uterine cross‐talk, especially during the implantation (Bidarimath & Tayade, 2017). The miRNAs are non‐coding, genetically transcribed, small molecules that fine‐tune the protein regulation, post‐transcriptionally (Gottesman, 2004). They are among the novel regulators of gene expression in the foetal and maternal tissues not only during conception but also throughout the pregnancy (Bidarimath and Tayade, 2017, Laresgoiti‐Servitje, 2015). The pregnancy‐associated miRNAs are implicated in numerous processes such as angiogenesis, trophoblast differentiation and the regulation of the maternal immune system (Laresgoiti‐Servitje, 2015, Lycoudi et al., 2015, Ali et al., 2021).

Buffalo is the predominant dairy animal in Southeast Asia which however, suffers from various reproductive constraints, for example, extended calving interval, late puberty, high incidence of anoestrous, implantation failures and silent oestrus (Zicarelli et al., 2007). High reproductive efficiency is an essential prerequisite for realising the optimum lifetime production potential of buffalo. A precise and early pregnancy diagnosis is a challenging task, which nonetheless is crucial for better reproductive management in buffaloes. The pregnancy‐associated miRNAs have also emerged as ideal candidates for early detection of pregnancy. The miRNAs hold significant promise as biomarkers in clinical settings since an altered expression of miRNAs has been reported in pregnancy complications (Lycoudi et al., 2015). A better understanding of the role of embryo‐derived miRNAs in pregnancy establishment would elucidate the potential of miRNAs to act as biomarkers for pregnancy detection. . Despite their importance, the role of pregnancy‐associated miRNAs in embryo implantation has not been elucidated, especially in livestock species like buffalo. Although many miRNAs are ascribed either to healthy pregnancies or pregnancy complications, there remains a lack of information regarding their target genes and the temporal behaviour of different miRNAs (Ali et al., 2021). Specifically, our understanding of the role of embryo‐specific miRNA in buffalo pregnancy is still in infancy and very limited information about their molecular function in blastocyst adherence during implantation is available. Although implantation marks the establishment of pregnancy and occurs between the Days 19 and 21 post fertilisation, nonetheless, to enhance the factors implicated in embryonic survival, decoding of the maternal‐foetal communication and interactions post fertilisation and implantation are equally important. For instance, a recent study by Valadão et al. (2018) reported that along with implantation, placentation is equally necessary to provide the embryo with nutrients and oxygen and therefore its survival. Besides, it has previously been reported that the trinucleated trophoblasts release secretory molecules such as microRNAs and proteins during placentation to maintain the foetal survival.

The present work was designed to analyse the temporal expression profile of cultured trophectoderm‐secreted miRs to identify the most abundant miR and determine its functional significance in implantation. We hypothesised that the trophectoderm secreted miRNAs assist in the endometrial remodelling and initiation of the MRP process, thus emerging as the earliest signals for the establishment of a successful pregnancy. Furthermore, the transfection of endometrial epithelial cells with the miRNA mimic would elucidate the signalling pathways and the molecules implicated in implantation and pregnancy establishment.

## 2 MATERIAL AND METHODS

All chemicals, media and reagents were procured from Sigma Aldrich Chemical Co. Ltd (USA), unless stated otherwise. The 0.22 μm filters were procured from Millipore Corporation, Bedford, MA, USA. The plasticware was obtained from Nunc (Thermo Scientific, USA). The foetal bovine serum (FBS) was obtained from Hyclone, Canada.

### 2.1 Trophectoderm cell culture and spent media collection

The collection of buffalo ovaries, oocyte aspiration and searching, in vitro maturation, fertilisation, and culture were done as described by Jain et al. (2016). The produced blastocysts were subsequently used for culturing the trophectoderm cells (TE cells). The TE cells were cultured following the method described by Mohapatra et al. (2015). Briefly, a group of four blastocysts per in vitro culture (IVC) medium drop (100 μl) was allowed to hatch and was seeded on the cumulus bed which was left undisturbed for 5 days to allow attachment. The IVC medium was replaced on the 6th day with the TE culture medium that consisted of DMEM with 20% FBS, 2 mM glutamine, 50 μg/ml gentamycin sulphate, 1% NEAA, and ITS (Insulin‐Transferrin‐Selenium). For miRNA isolation, 50 μl of the spent media was collected from each of the IVC drops (replaced with 50 μl fresh TE media) in 200 μl Eppendorf mini centrifuge tubes containing 50 μl lysis buffer (Qiazol) on Days 0, 6, 12, 16 and 21 (*N* = 3) of the trophectoderm (TE) cell culture and stored at –80°C, till further use. To establish the TE‐cell origin of microRNAs (rather than cumulus cells’), the spent medium collected from the cultured cumulus cells at Day 0 was used as a control.

### 2.2 Characterisation of trophectoderm cells using immunocytochemistry

After the termination of culture, the cultured TE cells were gently washed with 200 μl PBS for 5 min in 24‐well plates and subsequently fixed with 4% paraformaldehyde for 10 min at RT. The cells were then washed thrice with PBS and then treated with 1% Triton X‐100 for 10 min for permeabilisation. The cells were once again washed with PBS and blocked with 1% BSA (in DPBS) for 30 min at 38^°^C. Afterwards, 200 μl primary anti‐mouse antibody‐Cytokeratin18 and TE cell‐specific CDX2 were added at a 1:100 dilution (prepared in 1% BSA) and incubated overnight at 4°C. The cells were then washed with PBS thrice and incubated with FITC‐conjugated anti‐mouse IgG secondary antibody at 1:500 dilution in dark for 1 h at RT. The cells were washed three times with PBS and subsequently stained with nucleic acid stain Hoechst 33342 for 10 min at RT. Finally, the cells were washed with PBS and the micrographs were acquired on a BX‐51 microscope (Olympus). A non‐primary antibody control and the cumulus cells were used as a negative control since CDX2 is known to be absent in cumulus cells (Goissis & Cibelli, 2014).

### 2.3 Isolation of miRNAs from spent medium and cDNA synthesis

The secretory miRNAs was isolated from the spent culture media on Days 7, 12, 16 and 21 of the TE cell culture, as mentioned earlier using the miRNeasy Serum/Plasma Kit (QIAGEN) as per the manufacturer's instructions. As mentioned earlier, to confirm that secretory miRNAs have not originated from cumulus cells, we collected the media on Day 0 of the culture containing only the cumulus bed and this medium (Day 0) was considered as the control. The isolated miRNA was quantified using a NanoDrop ND‐1000 UV–Vis spectrophotometer (NanoDrop Technologies Inc., Wilmington, DE, USA). The miScript II RT kit (QIAGEN) was used for cDNA synthesis as per the manufacturer's instructions. Briefly, 5× Hispec buffer (4 μl), 10× nucleic mix (2 μl), water (2 μl), reverse transcriptase enzyme (2 μl) and 10 μl of miRNA (50 ng) were used to make up the final reaction volume of 20 μl. The mixture was incubated at 37°C for 60 min and at 95°C for 5 min to inactivate the miScript reverse transcriptase.

### 2.4 Real‐time PCR for miRNA quantification

The RT‐qPCR was carried out to elucidate the expression pattern of the selected panel of miRNAs (Table 1) in the TE cell culture spent media collected at various time points. The reaction mixture consisted of 12.5 μl of 2× miScript SYBR Green, 2 μl of cDNA template and 2.5 μl of primers for respective genes and 2.5 μl of 10× universal primer. The final reaction volume was made 25 μl with nuclease‐free water. The assay was performed in duplicates for each sample. The thermal profile was 95°C for 90 s, followed by 40 cycles consisting of denaturation at 94°C for 15 s, annealing at 55°C for 30 s and extension at 70°C for 30 s. The obtained *C*_q_ (cycle of quantification) values were normalised using an endogenous control (U6 snRNA) to assess the level of differences in total miRNA concentrations between the samples. The mean sample *C*_q_ values for different miRs were calculated for each sample from duplicate wells using the ΔΔCt  (Cycle threshold) method (Livak and Schmittgen, 2001). A no‐template control (NTC) was run on each plate to confirm the absence of nucleic acid contamination. To ensure the quality of RT‐qPCR and RT‐qPCR data MIQE guidelines were followed at every step (Bustin et al., 2009).

**TABLE 1:** The miRNAs selected for expression pattern analysis

miRNA	Species	Function	Stage	Reference	Primers
miR‐1246	Buffalo	Unknown	Peri‐implantation	Novel	F: AATGGATTTTTGGAGCAGG; R: miScript Universal Primer (sequence is proprietary)
miR‐let‐7a miR‐let‐7b	Mouse	Modulate mucin1 expression during implantation.	Peri‐implantation	Wilasinee et al.	F: TGAGGTAGTAGGTTGTATAGTT; R: miScript Universal Primer (Sequence is proprietary) F: TGAGGTAGTAGGTTGTGTGGTT; R: miScript Universal Primer (sequence is proprietary)
miR‐17‐5p	Bovine	Endometrial receptivity	Peri‐implantation	Attia Fatima et al.	F: CAAAGTGCTTACAGTGCAGGTAGT; R: miScript Universal Primer (sequence is proprietary)
miR‐126‐5p	Porcine	Endothelial cells proliferation	Peri‐implantation	Bidarimath et al.	F: CATTATTACTTTTGGTACGCG; R: miScript Universal Primer (sequence is proprietary)
miR‐26a	Porcine	Regulates the gene encoding cytokines such as Il6, Il6‐1R, PTGS2, PTGES etc.	Day 16 to Day 20	Krawczynski et al.	F: TTCAAGTAATCCAGGATAGGCT; R: miScript Universal Primer (sequence is proprietary)
miR‐125b	Bovine	Helps in maintaining pregnancy by immuno‐modulation	Day 16 to Day 20	Siristatidis et al.	F: TCCCTGAGACCCTAACTTGTGA; R: miScript Universal Primer (sequence is proprietary)

### 2.5 Endometrial epithelial cell isolation and culture

The buffalo uteri were collected aseptically from the Delhi abattoir and were brought to the lab on ice within 3 h of slaughter. The uteri were initially washed with 70% ethanol and then with 1× PBS twice. Afterwards, the uterine horns were filled with digestion media (100 ml 1% DPBS containing 0.08% trypsin, 1 ml penicillin‐streptomycin, and 100 mg BSA), tied using a sterile thread and dipped in 1× PBS in a beaker and kept at 37°C for 1 h. Later, the horns were untied and the digestion media were collected in a 50 ml tube. The digestion media containing cells after tissue digestion were pelleted down by centrifugation at 5000 rpm for 10 min then washed with 1×‐PBS and seeded in a tissue culture flask (T‐25) containing 3–5 ml of culture media (DMEM, NEAA, L‐Glutamine, penicillin‐streptomycin, and gentamycin). The cells were incubated for 18 h at 38.5°C in 5%CO_2_ and 95% humidity. Thereafter, the stromal cells were observed for adherence whilst the epithelial cells were collected from suspension and subsequently seeded in gelatin‐coated (0.1%) 48 well plates. The media was replaced after every two days at the 80% confluence stage.

### 2.6 Characterisation of isolated endometrial epithelial cells (EECs)

The endometrial epithelium cells (EECs) were cultured up to Day 6 and subsequently, these EECs were gently washed with 200 μl PBS for 5 min in 24‐well plates and then fixed in 4% paraformaldehyde for 10 min at RT. The cells were then washed with PBS thrice and subsequently treated with 1% Triton X‐100 for 10 min for permeabilisation. The cells were again washed with PBS and blocked with 1% BSA (in DPBS) for 30 min at 38°C. Next, a 200 μl primary anti‐mouse antibody, cytokeratin18 (Invitrogen) was added at a 1:20 dilution (prepared in 1% BSA) and incubated overnight at 4^°^C. The cells were then washed thrice with PBS and incubated with FITC conjugated anti‐mouse IgG secondary antibody (Invitrogen) at 1:20 dilution in dark for 1h at RT. The cells were washed three times with PBS and subsequently stained with Hoechst 33342 (molecular probes) with a working concentration of 1 μg/ml for 10 min at RT to stain the nuclei. Finally, the cells were washed with PBS and the micrographs were acquired on a BX‐51 microscope.

### 2.7 Priming of endometrial epithelial cells using interferon tau

The cultured endometrial epithelium cells (EECs) were treated with interferon tau (IFN‐τ) for preparing these cells for implantation by mimicking the in vivo priming of the endometrium by interferon tau, as reported in buffaloes previously (Kimmins et al., 2003). The IFN‐τ is a major cytokine produced by the peri‐implantation trophectoderm which contributes to the prevention of luteolysis by attenuating prostaglandin F2α (PGF) secretion from the uterine endometrium, resulting in pregnancy recognition and establishment (Kimmins et al., 2003). Briefly, the 200 μl of recombinant IFN‐τ (Cloud‐clone Corp.) was added in culture media at a concentration of 1 μg/ml to the cultured EECs in 24‐well plates after 48 h of culture.

### 2.8 Transfection of the mirVana™ miRNA mimic in cultured endometrial epithelial cells using Lipofectamine

The microRNA functional analysis was performed using a custom‐made mirVana™ miRNA mimic for the miR‐1246 (hsa‐miR‐1246, Assay ID: MC13182, miRBase version v 22.0). This micro‐RNA was selected based on the results of NGS data in our lab that indicated its highest abundance on Day 21 (Sarwalia et al., 2021, NCBI, Accession: PRJNA705293, ID: 705293). Besides, it is a novel miR whose role in MRP has not been hitherto elucidated. The transfection was performed in the cultured EECs on Day 7 when the cells were nearly 80% confluent. The EEC culture medium was replaced by serum‐free opti‐MEM media 2 h before transfection. The RNA Imax lipofectamine reagent (4 μl, Invitrogen, USA) and miRNA‐1246 mimic (15 μl, 30 nmol) were diluted in 100 μl opti‐MEM in an RNase free microcentrifuge tube and incubated for 15 min at 37°C. Subsequently, 200 μl of the lipofection solution was added to each well of 24‐well plates and incubated for 4 h after which the medium was replaced by the EEC culture media.

### 2.9 Gene‐target prediction of the selected miRNAs

The target prediction of the selected miRNAs miR‐1246 was performed using three different bioinformatics tools: DIANA‐TarBase v7.0, miRDB v5.0 and Target Scan v7.2. The TarBase is a reference database for manually curated experimentally tested and validated miRNA: gene interactions for multiple species which provides a comprehensive data set that assists in easily finding targets for miRNAs (Karagkouni et al., 2018). The miRDB database is used not only for miRNA target prediction but also for functional annotations by identifying the common features associated with miRNA binding and target downregulation (Weijun Liu and Xiaowei Wang, 2019). The Target Scan tool uses an improved quantitative model of canonical targeting which used multiple features, for example, sequence complementarity, presence of conserved 8mer, 7mer and 6mer sites that match the seed region of each miRNA and other statistical parameters to predict the most effectively targeted mRNAs (McGeary et al., 2019). Apart from these indirect prediction methods, we directly selected the miRNA targets based on manually determined sequence complementarity and those from published literature. Next for the quantitative reverse transcription PCR (RT‐qPCR), the total RNA was isolated from cultured/transfected endometrial epithelial cells using the Trizol method after 24 h of transfection. Briefly, 800 μl of Trizol reagent was added and 200 μl of chloroform was added and mixed. The samples were centrifuged at 12,000 rpm for 10 min at 4°C. After centrifugation, the uppermost aqueous layer was taken and an equal volume of isopropanol was added and incubated at 4°C for 5 min and samples were centrifuged at 12,000 rpm for 15 min at 4°C. The resuspended pellet was washed twice with 70% ethanol at 10,000 rpm for 10 min at 4°C. The air‐dried RNA pellet was dissolved in 25 ml of DEPC treated nuclease‐free water. Total RNA was quantified using a Nanodrop spectrophotometer and a fixed amount of 1 μg RNA was used for cDNA synthesis using PrimeScript™ 1st strand cDNA Synthesis Kit (Clontech, Takara) following the manufacturer's instructions. The relative quantification of target genes was done using Maxima SYBR Green qPCR Master Mix (Fermentas, USA) in a final reaction volume of 10 μl, as described by Batra et al. (2019). The RT‐qPCR conditions were 95˚C for 10 min, followed by 40 cycles of denaturation at 95˚C for 15 s, annealing at 59˚C for 15 s and extension at 72˚C for 20 s, followed by the melting curve protocol with 10 s at 95˚C and then 60 s each at 0.5˚C increments between 65˚C and 95˚C. The reactions were performed in duplicate for each sample. The relative amounts of target gene expression for each sample were calculated using the formula 2−(ΔΔCT) against an endogenous control RPS18 for genes. A no‐template control (NTC) was run on each plate to confirm the absence of nucleic acid contamination. To ensure the quality of RT‐qPCR and RT‐qPCR data, MIQE guidelines were followed at every step (Bustin et al., 2009).

### 2.10 Statistical analysis

The relative expression of miRNAs (during different days of TE culture) and genes (under various conditions) was examined for normality of distribution. The Brown‐Forsythe test, as implemented in GraphPad, was used to test the differences in the standard deviations of the miRNA and gene expression. The Brown‐Forsythe test was found to be non‐significant for all the sample groups. The data were analysed by ANOVA and Tukey's post hoc test as implemented in GraphPad Prism 7.0 (for Windows, GraphPad Software, La Jolla California USA, www.graphpad.com) and a *p*‐value <0.05 was considered to be statistically significant.

## 3 RESULTS

### 3.1 Primary culture of buffalo trophectoderm cells and their characterisation

**FIGURE 1:**
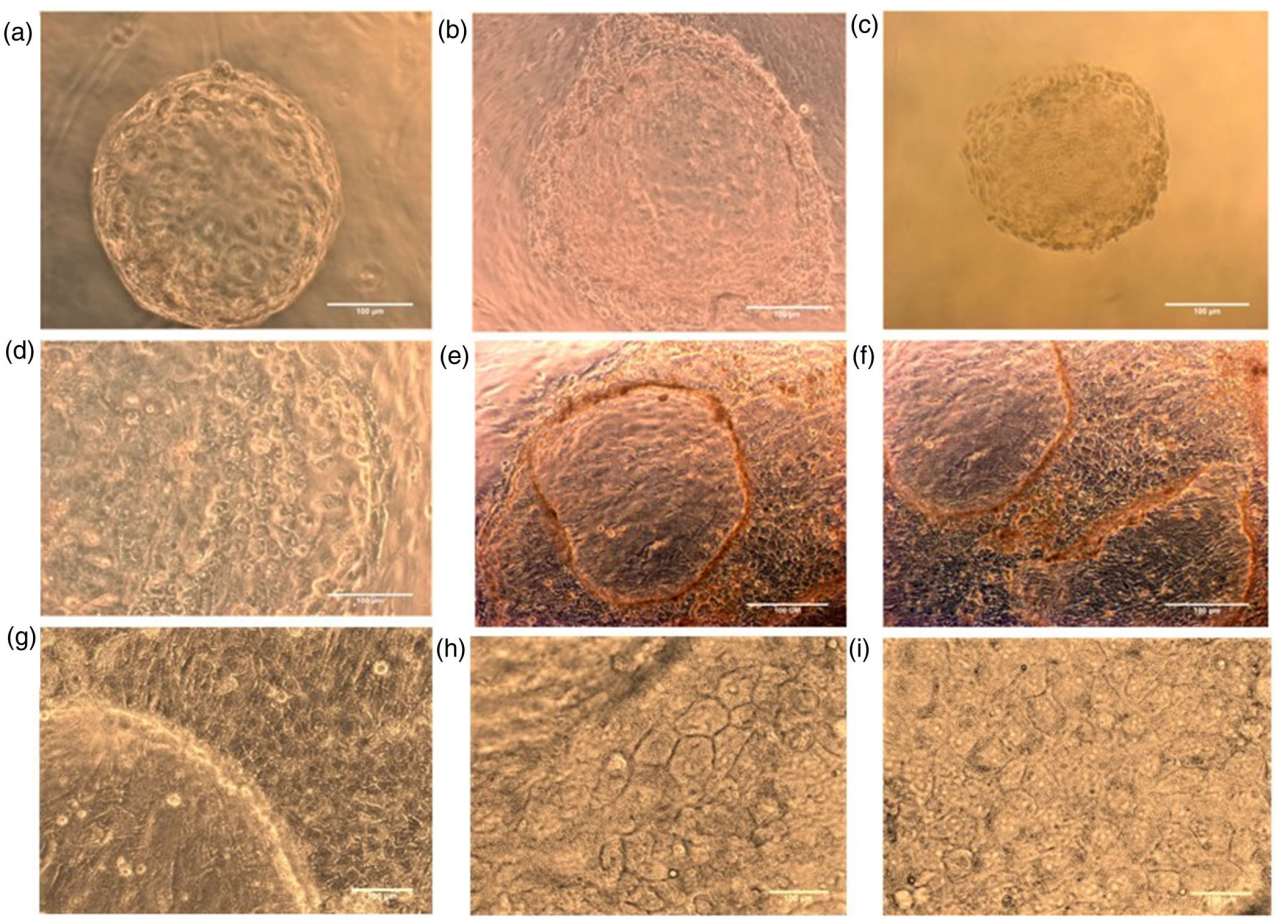
Trophectoderm cell culture: (a) Hatched blastocyst seeded onto the cumulus feeder layer on Day 9 of IVF. (b) Blastocysts initiating attachment to the feeder layer – Day 6. (c) Fully attached blastocyst on the cumulus bed – Day 8. (d) Trophectoderm cells exhibiting their characteristic cuboidal morphology with a centrally located large nucleus – Day 12. (e) TE cells started vesicle formation by Day 17. (f) TE cells at Day 21. (g) Cuboidal shaped TE cells. (h) TE cells showing dark granules in cytoplasm. (i) Binucleated TE cells, syncytiotrophoblast.

**FIGURE 2:**
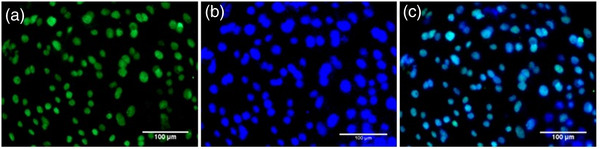
Characterisation of the cultured TE cells. (a) The purity of the cultured TE cells was confirmed by the presence of TE‐specific marker CDX2 wherein the green fluorescence specific to CDX2 could be observed in the cultured trophectoderm cells. (b) Blue fluorescence shows nucleus stained with Hoechst. (c) Merged.

An in vitro model for buffalo TE cell culture was established wherein the buffalo TE cells were grown and maintained till Day 21 of the culture (Figures 1 and S1). The primary culture of these cells was established by culturing the hatched blastocysts on the mitomycin‐C inactivated cumulus feeder layer (Figure 1a). The TE cells adhered to the cumulus feeder layer by Day 6 (Figure 1b) and were fully attached by Day 8 (Figure 1c). The TE cells were easily distinguishable from either the ICM or the cumulus cells because of their characteristic cuboidal morphology with a centrally located large nucleus (Figure 1d). These cells exhibited their hallmark trait of vesicle formation on Day 17 by the end of the culture (Figure 1e). Moreover, the cell cytoplasm revealed typical granularisation, the presence of lipid droplets, and the differentiation of trophectoderm cells to syncytio‐trophectoderm characterised by the presence of two to three nuclei per cell (Figure 1g–i). These characteristic features of the cultured buffalo TE cells confirmed the purity of these cells (Mohapatra et al., 2015). The purity of the cultured TE cells was further confirmed by the presence of TE‐specific marker CDX2 wherein the green fluorescence specific to CDX2 could be observed in the cultured trophectoderm cells (Figure 2).

### 3.2 The novel candidate bta‐miR‐1246 along with miRs miR‐let‐7a and miR‐let‐7b demonstrated the most dynamic expression pattern during the window of implantation

**FIGURE 3:**
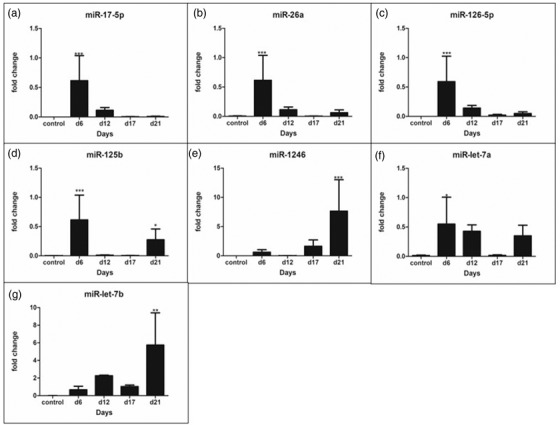
Expression analysis of TE secreted miRNAs. The gene expression for a panel of miRNAs was profiled over a period of 21 days and Day 0 was considered as the calibrator. The significance of differences between means was calculated at a 5% level (*p* < 0.05).

Among the seven miRs selected for the expression dynamics in TE spent media, the miR‐1246 qualified as a novel candidate selected from our lab's NGS data (Sarwalia et al., 2021, NCBI, Accession: PRJNA705293, ID: 705293) while the remaining six miRs were selected based on the previous literature (Inyawilert et al., 2015, Chen et al., 2016 Krawczynski et al., 2015; Siristatidis et al., 2015, Kaczmarek et al., 2020). The three miRs, namely, miR‐1246, miR‐let‐7a and miR‐let‐7b exhibited a very typical expression pattern on Day 21, the expected day of maternal recognition of pregnancy (MRP) (Figure 3) which corresponds to placentation in this experiment (Day 21+ 9 days of blastocyst formation). The miR‐1246 exhibited a sudden increase in their expression on Day 21 as compared to the other experimental days, that is, Days 6, 12 and 17. Along with miR‐1246, the miR‐let‐7b was also found to be highly expressed on Day 21 (*p* < 0.05). The expression pattern of miR‐1246, miR‐let‐7a and miR‐let‐7b indicated that they are crucial in the later stages of TE cell growth (Day 21) vis‐à‐vis the initial stages which suggests an important role during the window of implantation. Contrarily, the miR‐125b was found to be abundant in the initial stages and then it is expression was declined during the window of implantation. Likewise, the expression of miR‐17‐5p, miR‐26a and miR‐126‐5p was dominant during the initial stages of development and gradually diminished towards Day 21. This shows that these miRs akin to miR‐125b are playing role in the initial stage of TE cell growth and are downregulated subsequently.

### 3.3 Beta‐catenin, mucin, integrin beta‐3 and integrin beta‐8 were the predicted targets for miR‐1246

We employed both the direct targets (based on sequence complementarity) and indirect targets (based on in silico prediction) of miR‐1246 for determining their regulation by miR‐1246 during the implantation window. The target genes were also selected from previous literature in addition to being predicted de novo using various in silico target prediction tools. The genes LIF, LIFR and osteopontin (literature‐based) play an important role in implantation and exhibit very peculiar expression patterns during endometrial remodelling and assist embryo implantation. Although these genes are not the direct targets (based on sequence complementarity) for miR‐1246, nonetheless, they were considered for analysing the indirect regulation of these genes (as predicted as putative targets) by miR‐1246. The corresponding target hits from the three tools (Target scan, DIANA and miRDB) were filtered based on their predicted target score as well their relevance during implantation. The results for the target prediction for miR‐1246 are depicted in Table 1. These results revealed that out of all the predicted targets for bta‐miR‐1246, the mucin1, integrin beta‐8, integrin beta‐3 and beta‐catenin were the most likely targets for miR‐1246.

### 3.4 Primary culture of endometrial epithelial cells and their characterisation

**FIGURE 4:**
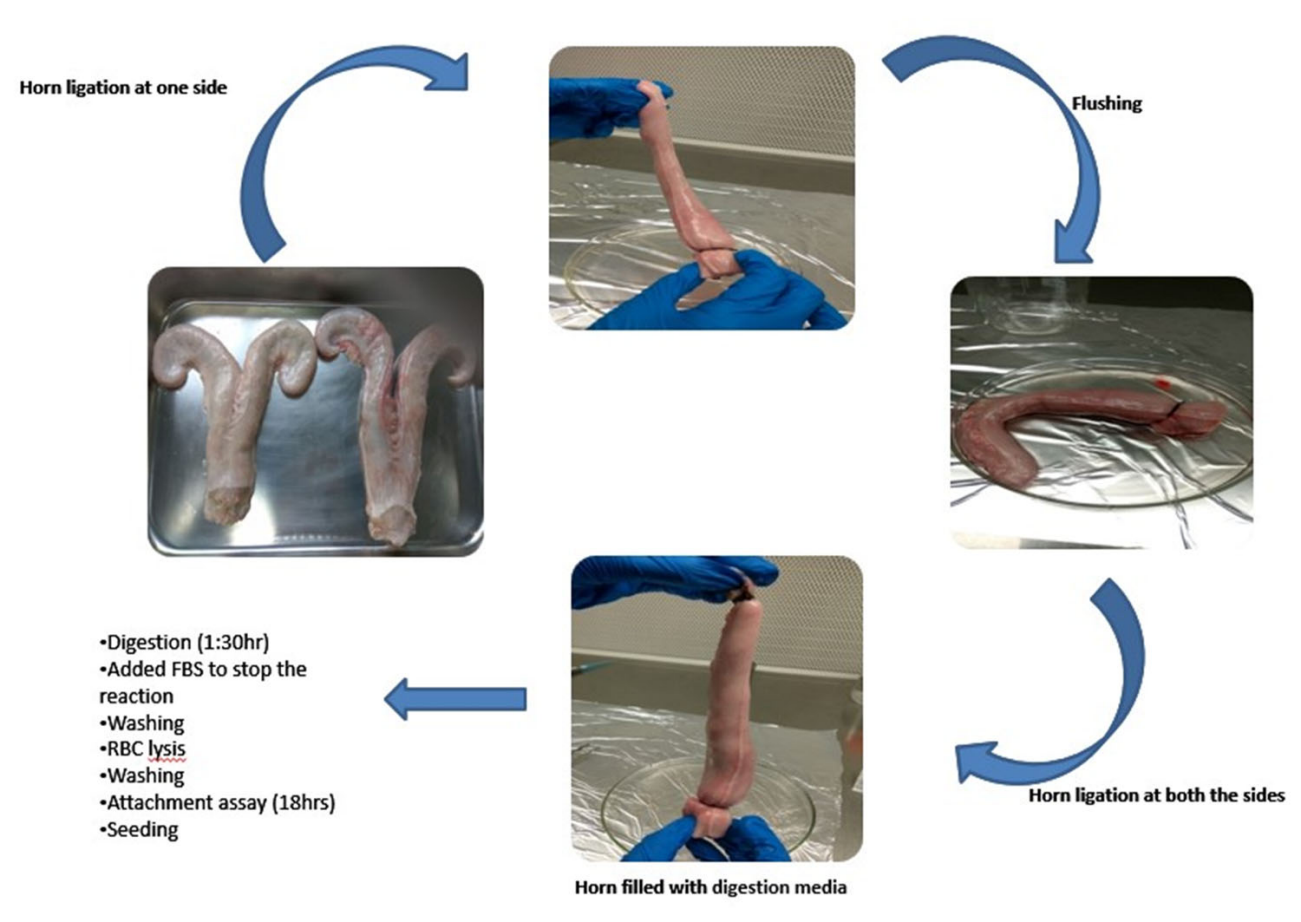
Endometrial epithelial cell isolation using the tubal ligation method

**FIGURE 5:**
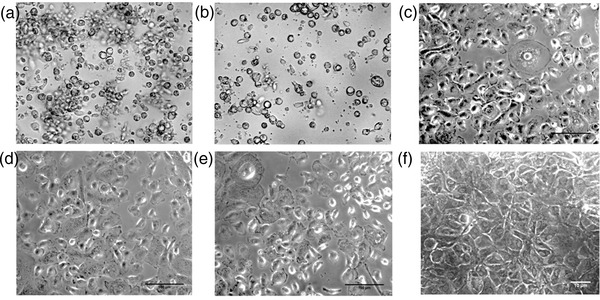
Endometrium Epithelial cell culture. (a) Isolated endometrium cell population on Day1 contained the desired endometrial epithelial and the contaminating stromal cells. (b) Pure, large spherical‐shaped endometrium epithelial cells (EEC) separated from the stromal cells by seeding the isolated cell population for 18 h on gelatin‐coated plates. (c) EEC changed their morphology from spherical to polygonal after Day 3 of culture (d) EEC on Day 12 (e) EEC on Day 17 (f) EEC on Day 21 reaching the confluency. All images were acquired at 200× magnification.

**FIGURE 6:**
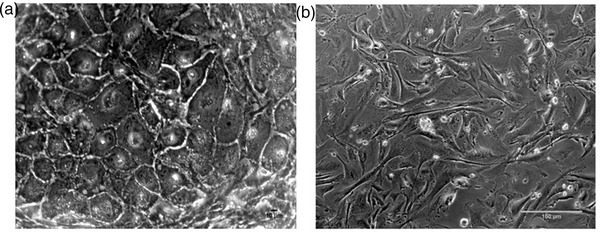
Morphological characterisation of Endometrium cells: (a) Polygonal shaped endometrial epithelial cells had granular cytoplasm with centrally located large nucleus, 400×. (b) The contaminating, spindle‐shaped stromal cells, 400×.

**FIGURE 7:**
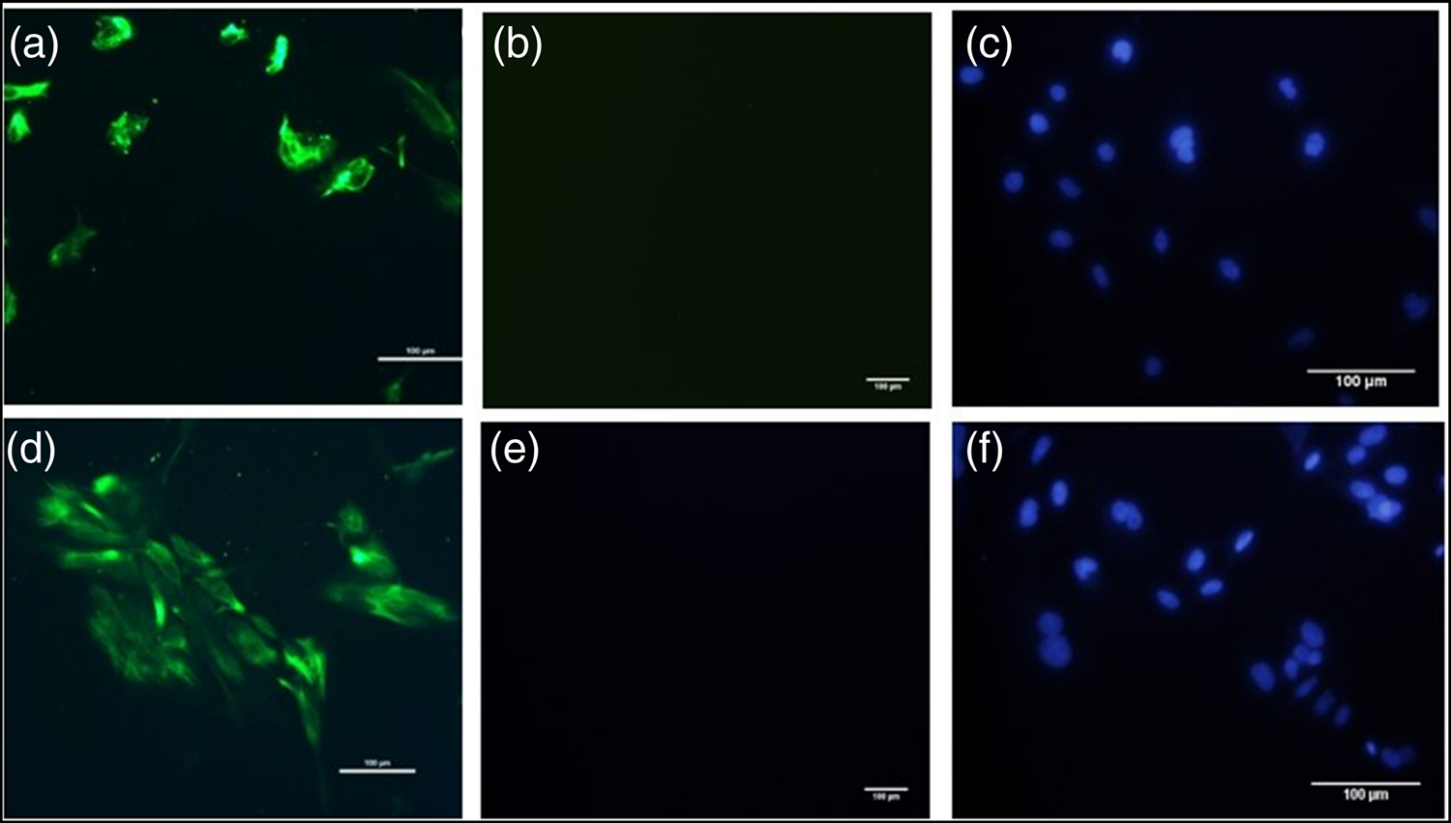
Characterisation of Endometrium cells: (a) Green fluorescence shows the cytokeratin expression in epithelial cells. (b) Purity of EECs was checked by cytokeratin staining in stromal cells (negative control). (c) Hoechst staining for EEC nuclei. (d) Green fluorescence shows vimentin expression in stromal cells. (e) EECs were stained with vimentin as a negative control. (f) Hoechst staining for stromal cell nuclei.

The buffalo endometrial epithelial cells (EECs) were grown in vitro and maintained till Day 21 of the culture. The peculiar characteristics of the cultured EECs (discussed below) assisted in distinguishing these cells from the contaminant stromal cells. The primary EEC culture was established by isolating epithelial cells using the tubal ligation method, the highest cell yield being observed at 0.08% trypsin (Figure 4). The morphological progression of endometrial epithelial cells up to Day 21 is depicted in Figure 5. The isolated cell population contained the desired endometrial epithelial and some contaminating stromal cells (Figures 5 and 6). Nonetheless, seeding the isolated cell population for 18 h in a culture flask could effectively separate the pure epithelial cells from the stromal cell population (Figures 5 and 6). The large spherical‐shaped epithelial cells remained in the suspension whilst the stromal cells were attached to the substratum (culture flask) after 18 h. The separated epithelial cells were then seeded on 0.1% gelatin‐coated 24‐well culture plates wherein these cells were observed to attach to the substratum by Day 2 (Figure 5b). The EECs changed their morphology from spherical to polygonal by Day 3 of culture (Figures 5 and 6) and gradually started attaining confluence which peaked at Day 21 (Figures 5 and 6). As observed in Figure 6, the EECs exhibited a granular cytoplasm with a centrally located large nucleus. These characteristic features of EECs were distinguishable from the spindle‐shaped endometrial stromal cells. Further, the purity of epithelial cells was determined by antigenic characterisation using immunocytochemistry (ICC). The vimentin and cytokeratin18 are the two characteristic markers for endometrial stromal cells and endometrial epithelial cells, respectively which were used to screen the cultured epithelial cells. The cytokeratin expression was observed specifically in epithelial cells, not in stromal cells whereas the expression of vimentin was only observed in stromal cells (Figure 7).

### 3.5 The endometrial epithelium receptivity genes, namely, beta‐catenin and mucin were downregulated after transfection of EECs with miR‐1246 mimic

**FIGURE 8:**
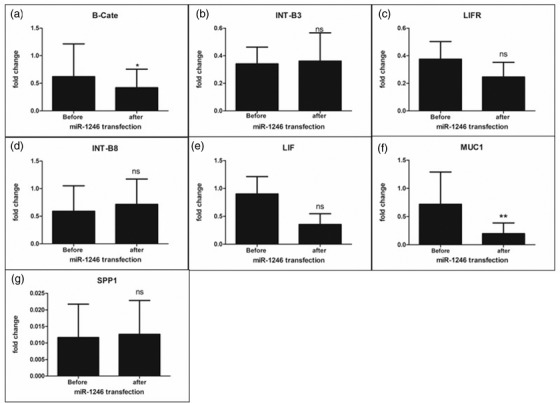
Expression analysis of endometrium receptivity genes. The gene expression for a panel of target genes was profiled before and after transfection. The MUC1 and beta‐catenin were down significantly regulated after miR‐1246 transfection of endometrial epithelial cells. The expression in non‐primed EECs was considered to be the calibrator. The significance of differences between means was calculated at a 5% level (*p* < 0.05).

**FIGURE 9:**
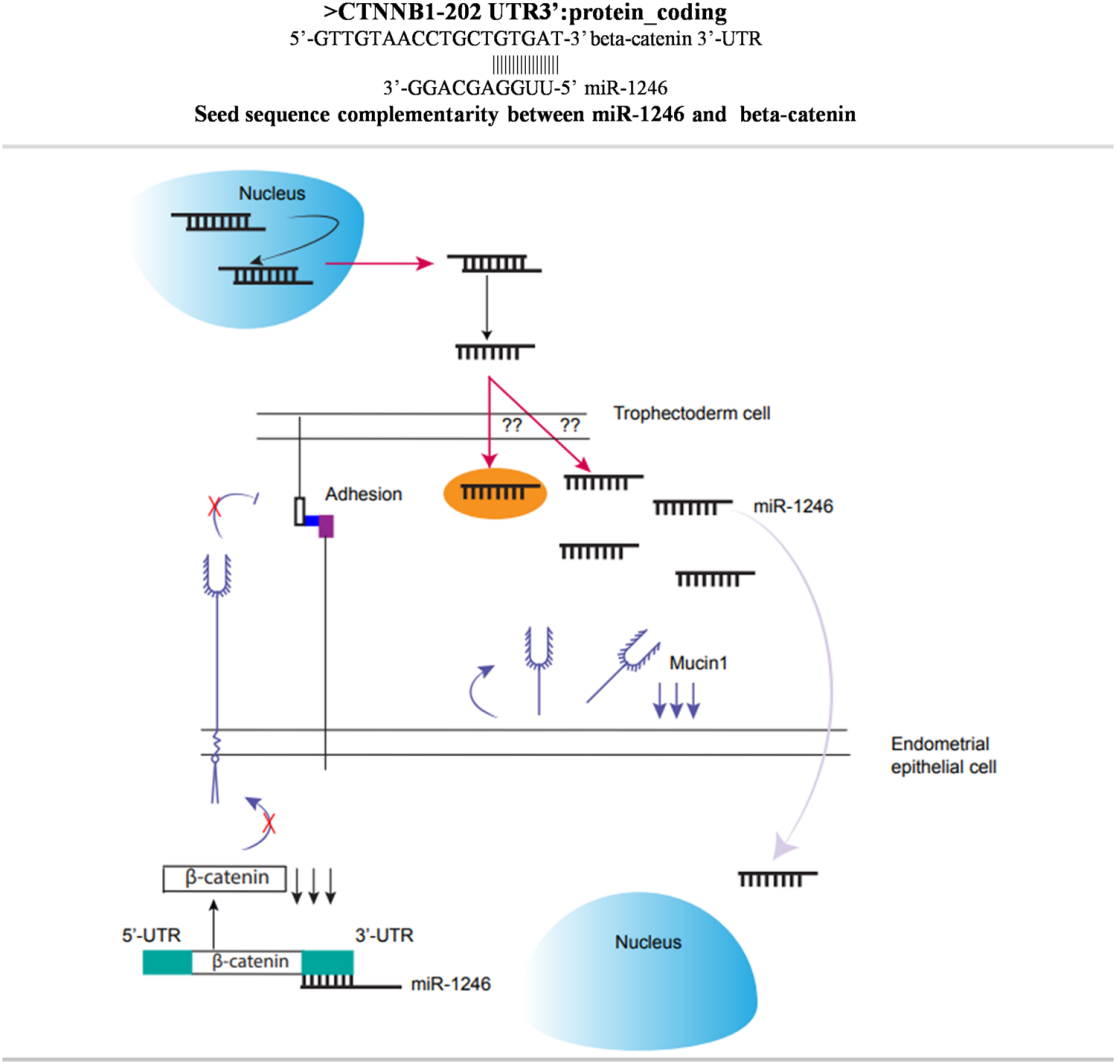
(a) Complementarity between the 3’UTR of beta‐catenin and seed sequence of miR‐1246. (b) Mechanism of action by miR‐1246. The miR‐1246 downregulates the expression of beta‐catenin and mucin1 mRNAs

Among the seven target genes, namely, integrin beta‐3 (INT‐B3), integrin beta‐8 (INT‐B8), mucin (MUC1), osteopontin (spp1), beta‐catenin, LIF and LIFR, the expression of only the direct targets (explained below) of miR‐1246, that is, mucin and beta‐catenin was found to be significantly downregulated after transfection of EECs with miR‐1246 mimic. The standardisation of transfection was done using the pAcGFP1‐N1 vector (Supplementary Figure S2). The transfection EECs with miRNA‐1246 mimic significantly decreased (*p* < 0.05) the expression of mucin1 and beta‐catenin in the transfected cells vis‐à‐vis control, that is, the primed EECs before transfection (Figure 8). Notably, the seed sequence analysis revealed that the seed sequences for the miR‐1246 3’‐GGACGA‐5’ and 5’‐AATGG‐3’ are complementary to the 3’ UTR sequence of beta‐catenin, 5’‐CCTGCT‐3’ and 3’UTR sequence of muc1 3’‐TTACC‐5’, respectively (Figure 9). This explained the transcriptional downregulation of beta‐catenin and mucin exerted by miR‐1246 through direct sequence complementarity. No significant change, however, was observed in the expression of SPP1, LIF, LIFR INT‐B3 and INT‐B8 expression as a result of the miR 1246 mimic transfection.

## 4 DISCUSSION

The present work was undertaken to identify the most abundant miRNAs secreted from the buffalo trophectoderm cells and elucidate their role in implantation (Day 12, in this experiment) and placentation (Day 21). The miRNAs have recently been discovered to be the novel molecular candidates involved in embryo‐uterine cross‐talk; however, the expression dynamics of the pregnancy‐associated miRNAs and their molecular function at the cellular level are not well understood. We wanted to reveal the molecular dialogue between the embryo and the endometrium which is involved in the establishment of a successful pregnancy in buffalo. Our results indicated that the trophectoderm cells maximally express miR‐1246, let‐7a and let‐7b during the window of implantation (Day). Apparently, the novel miR‐1246 is involved in the endometrial remodelling by regulating the expression of the two important implantation‐associated genes, that is, beta‐catenin and mucin1. These results underpin the importance of miR‐1246 which could also be used as an MRP marker during the early stages of pregnancy in buffalo.

The embryo‐uterine cross‐talk through miRNAs can be elucidated by establishing an in vitro MRP model consisting of trophectoderm cell culture and endometrium epithelial cell culture. We established an MRP model in buffalo by culturing the in vitro produced embryos on a cumulus cell feeder layer. The growth of the TE cells varies depending upon the feeder layer used and it has been reported that the cumulus feeder layer enhances the growth of TE cells in bovids (Saadeldin et al., 2017). The detailed ultra‐structural appearance of TE cells in humans has earlier been reported (Ahlström et al., 2011) wherein the best‐cultured TE cells (scored by their number and cohesiveness) reportedly contained many cells that form a cohesive epithelium, as observed in this study (Figure 2). The grading of the TE cells is associated with the implantation efficiency of human embryos (Ahlström et al., 2011). Besides, the purity of TE cells was confirmed by the TE‐specific markers CDX2 and cytokeratin 18 (Hou et al., 2015). CDX2 has been used as a specific marker that is capable of distinguishing between TE and ICM cells in mice, porcine and other animals (Hou et al., 2015). The CDX2 expression observed in the buffalo TE cells derived from IVF blastocysts in this study was found to be similar to the expression patterns previously reported in cattle, porcine and mouse embryos (Fujii et al., 2010; Berg et al., 2011). Similarly, the cytokeratin 18 (CK18) is solely expressed in the trophectoderm and can therefore be used as a marker for trophectodermal differentiation (Adjaye et al., 2007; Goossens et al., 2010).). The buffalo IVF blastocyst‐derived TE cells stained positive for CDX‐2 and cytokeratin 18 which confirmed the purity of these cells.

The remaining fraction of the buffalo MRP model was developed by isolating the epithelial cells from the buffalo uterus and culturing the isolated endometrial epithelial cells (EECs) on the gelatin‐coated culture plates which helped in the three‐dimensional growth of the EECs. We observed that initially, the EECs were spherical in shape (Figure 5), which nevertheless transformed to a polygonal shape with a large centrally located nucleus depicting the peculiar characteristic of endometrial epithelial cells, as described previously in humans (Li et al., 2018). These cells have been reported to attain a polyhedral shape when cultured on a plastic stratum; however, the deterioration of cell integrity was observed within 2 weeks of culture which is marked by the appearance of vacuoles in the cytoplasm (Arnold et al., 2001). On the contrary, the buffalo EECs were found to maintain their characteristic morphology and were in the proliferative state even after 21 days of culture. The use of gelatin coating appeared to help in the maintenance of cell morphology and integrity which is known to provide a scaffold that assists in the three‐dimensional growth of cells.

Recent studies have revealed that miRNAs can be released by cells into the extracellular environment facilitating intercellular communication and providing indicative information associated with physiological and pathological conditions (Ali et al., 2021; Krawczynski et al., 2015; Siristatidis et al., 2015, Kaczmarek et al., 2020). The microRNAs (miRs) which function as the transcriptional regulators of gene expression have been widely reported to be involved in embryo implantation (Dior et al., 2014). The discovery of extracellular miRNAs has shed new light on implantation associated changes and has revealed novel mechanisms for embryo‐maternal communication (Liang et al., 2017). Moreover, they may serve as non‐invasive biomarkers for the assessment of endometrial receptivity with improved accuracy of evaluation while reducing the mechanical damage to the animal (Wang and Jiang, 2007; Liang et al., 2017). We considered seven TE secreted extracellular miRNAs for the embryo‐uterine cross‐talk which could be crucial during embryo implantation. The expression of TE secreted miR‐1246 was considerably high during the window of implantation indicating its involvement in the implantation process. This is a novel molecule involved in the remodelling of the endometrium, and to the best of our knowledge, no reports exist implicating it in the implantation process. Similarly, an upregulated expression was observed for the miR‐let‐7 family which is known to increase the uterine receptivity via inhibition of WNT signalling and also by suppressing anti‐adhesive, MUC1 in mice and humans (Inyawilert et al., 2015). We observed an increased expression of miR‐let‐7b on Day 21 of TE culture indicating its role in buffalo embryo implantation. Interestingly, the transfection by miR‐1246 suppressed the expression of MUC1 in buffalo, as reported for the miR‐let‐7 family (Inyawilert et al., 2015). Contrarily, the expression of buffalo miR‐125b was high during the initial stages and declined at the later stages of TE cell development indicating that its downregulation is crucial during implantation. Our results are in agreement with a recent report on humans wherein it has been demonstrated that miR‐125b triggers endometrium receptivity decline through the regulation of MMP‐26 function (Chen et al., 2016). The miR‐26a has been demonstrated to be secreted from trophoblast cells on Day 16 and involved in embryo‐maternal communication whereas we observed its highest expression on Day 6 in buffalo (Krawczynski et al., 2015; Siristatidis et al., 2015). Likewise, the expression of miR‐17‐5p and miR‐126‐5p which are implicated in embryo implantation in several species like humans, mice and pigs was highest on Day 6 in buffalo, as reported earlier (Kaczmarek et al., 2020). In the case of buffalo, we found higher expression of these miRs during the initial stages of TE cell development suggesting that in buffalo these miRs are more important in the initial stage of TE cell differentiation. Differential placentation could be the reason attributed for these anomalies since the placentation pattern of mice, sheep and pigs are characteristically different from that of the cattle and buffaloes (Lijie Su et al., 2014; Bazer et al., 2009; Green et al., 2021). It would be interesting to elucidate the molecular mechanism behind these species‐specific differences in miR expression dynamics.

Among the seven miRs, we selected miR‐1246 for transfection since (i) its expression in TE spent media was highest during implantation vis‐à‐vis remaining miRs; (ii) its function was unexplored in embryo implantation; and (iii) NGS data in our lab hinted towards its abundance during the period of implantation. Although MUC1 and beta‐catenin were among the direct targets for miR‐1246 based on complementarity (as confirmed by seed sequence analysis), we also chose to elucidate the expression of molecules known to be implicated in implantations, for example,, Integrin beta‐3, integrin beta‐8, osteopontin, LIF and LIFR. The LIF‐LIFR‐gp130 complex is known to make the endometrium receptive by activating the JAK‐STAT pathway (Rosario and Stewart, 2016). Our results indicated that both the LIF and LIFR were expressed in EECs on Day 6 of culture pointing towards their role in endometrial differentiation. Nevertheless, after transfection with miR‐1246 mimic, their expression was reduced to half of its original value. Our data suggested that the LIF and LIFR are being regulated by miR‐1246 indirectly albeit the effect was deemed statistically non‐significant. Likewise, we did not observe any significant change in the expression of integrin beta‐3, integrin beta‐8 and osteopontin (SPP) after the transfection of EEC cells by miR‐1246 mimic. However, we could confirm the expression of integrin beta‐3, integrin beta‐8, and osteopontin in the IFN‐τ primed buffalo EEC cells which could be important for endometrium receptivity in buffaloes.

As mentioned earlier, the expression of only the beta‐catenin and MUC1 was found to be significantly downregulated after miR‐1246 mimic transfection. It has earlier been reported that the beta‐catenin signalling pathway is inhibited in both the blastocyst and uterus during the window of implantation. This may represent a new mechanism to synchronise the development of preimplantation embryos and differentiation of the uterus during this process in mice (Li et al., 2005). Our data in buffalo suggested that downregulation of beta‐catenin by miR‐1246 could assist the endometrium in preparing for implantation. The MUC1 expressed on the endometrium is an anti‐adhesive glycocalyx that prevents the adhesion of embryos to the uterus. A reduction in MUC1 on uterine epithelial cells at the implantation site has been deemed crucial for successful implantation, as reported in mice (Wang et al., 2007). Only if the embryo is potent enough to downregulate the MUC1, it can attach to the endometrium (Dharmaraj et al., 2009). This leads to a possible mechanism whereby poor‐quality embryos, lacking the ability to cause downregulation of MUC1, might fail to implant (Meseguer et al., 2001). The downregulation of MUC1 is very specific and highly localised in mice and humans which guides the blastocyst to the precise area that is considered fittest for implantation (Wilasinee et al., 2015). Intuitively, a higher expression of MUC1 has recently been demonstrated to lead to impaired endometrium receptivity and decidualisation in mice and women with polycystic ovarian syndrome (PCOS) (Budihastuti et al., 2020). Thus, our data concur with the abovementioned reports and suggest the role of TE cell‐secreted miR‐1246 in remodelling the endometrium for implantation. Similar to miR‐1246, the miR‐let ‐7a and 7b have been reported to downregulate mucin expression in rodents which facilitates attachment of blastocysts on the endometrium (Wilasinee et al., 2015). Thus the role of the novel miR‐1246 in embryo implantation could be well contemplated akin to the already reported miR‐let ‐7a and 7b based on their gene expression profile and downregulation of common target gene (MUC1).

## 5 CONCLUSION

Our data demonstrated the temporal variations in the expression of a novel micro‐RNA, miR‐1246 during the course of trophectoderm maturation which exhibited the highest expression at Day 21 (the window of implantation). This miR could be implicated in early pregnancy establishment since it downregulated both of its direct targets MUC1 and beta‐catenin, a process that is crucial for blastocyst attachment during implantation of the embryo (Li et al., 2005). Further studies are required to address the mechanisms of synergistic communication between maternal and embryonic cells to elucidate the molecular mechanism behind the regulation of pathways involved in the maternal recognition of pregnancy.

## AUTHOR CONTRIBUTIONS

Pratiksha Dubey: data curation; formal analysis; investigation; writing – original draft; writing – review & editing. Vipul Batra: formal analysis; methodology; writing – original draft; writing – review & editing. Parul Sarwalia: data curation; formal analysis. Samiksha Nayak: visualisation. Rubina Baithalu: data curation; formal analysis. Rakesh Kumar: conceptualisation; resources; supervision.

## CONFLICT OF INTEREST

The authors declare that the research was conducted in the absence of any commercial or financial relationships that could be construed as a potential conflict of interest.

## FUNDING

Department of Biotechnology, New Delhi, India‐Funded project (Grant No. BT/PR14013/AAQ/1/629/2015) provided the funds for performing the experiments under this research work. National agricultural Science Fund Award No. NASF/ABA‐7005/2018‐19 provided the funds for the interpretation of the results generated using these experiments.

### ETHICAL STATEMENT

The authors confirm that the ethical policies of the journal, as noted on the journal's author guidelines page, have been adhered to and the appropriate ethical review committee approval (IAEC‐IACR, NDRI) has been received. The US National Research Council's guidelines for the Care and Use of Laboratory Animals were followed.

### PEER REVIEW

The peer review history for this article is available at https://publons.com/publon/10.1002/vms3.968.

## Supporting information



FIGURE S1: Cumulus cell culture
FIGURE S2: Transfection of EECs with GFP

## Data Availability

The data that supports the findings of this study are available in the supplementary material of this article.
