# Supplementary material for: miR‐1246 is implicated as a possible candidate for endometrium remodelling facilitating implantation in buffalo (Bubalus bubalis)
Source: Vet Med Sci. 2022 Oct 25;9(1):443–56. doi: 10.1002/vms3.968 (PMC9857007; doi:10.1002/vms3.968)
Supplement: Supplementary file 1 — FIGURE S1: Cumulus cell culture FIGURE S2: Transfection of EECs with GFP [file VMS3-9-443-s001.docx]

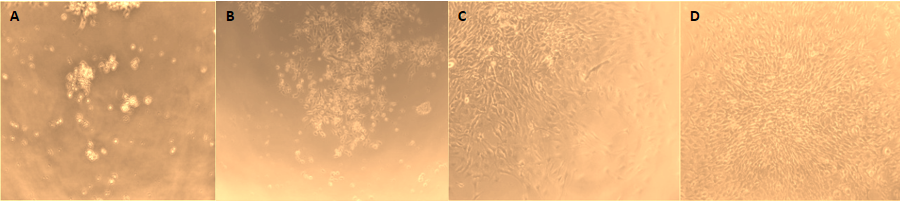


**Fig S1: Cumulus cell culture: A)** Cumulus cells on the day of culture, 10X **B)** day 6 **C)** day 12 **20X D)** day 18 at 200X magnification

**
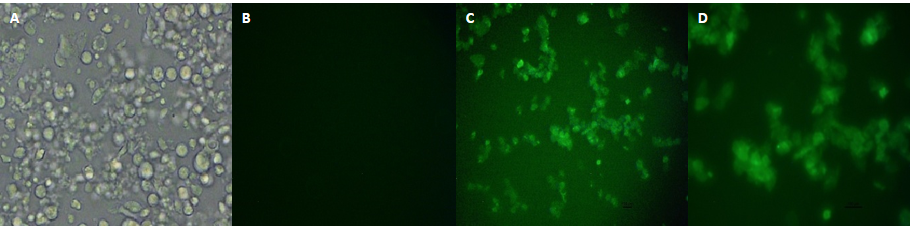
**

**FigS2: Transfection of EECs with GFP: A)** EECs in bright field **B)** negative control **C)** EECs after transfection at 100X magnification **D)** EECs after transfection at 200X magnification
